# The Senegal urban reproductive health initiative: a longitudinal program impact evaluation^☆^^☆☆^

**DOI:** 10.1016/j.contraception.2018.01.003

**Published:** 2018-05

**Authors:** Aimee Benson, Lisa Calhoun, Meghan Corroon, Abdou Gueye, David Guilkey, Essete Kebede, Peter Lance, Rick O'Hara, Ilene S. Speizer, John Stewart, Jennifer Winston

**Affiliations:** Carolina Population Center, University of North Carolina at Chapel Hill; IntraHealth International, Dakar, Senegal (Gueye)

**Keywords:** Senegal, Family planning, Urban, Evaluation, Longitudinal

## Abstract

**Objectives:**

This paper presents the impact of key components of the Senegal Urban Reproductive Health Initiative, including radio, television, community-based activities, Muslim religious-leader engagement and service quality improvement on modern contraceptive use by all women and the sub-sample of poor women.

**Study design:**

This study uses baseline (2011) and endline (2015) longitudinal data from a representative sample of urban women first surveyed in 2011 to examine the impact of the Initiative's demand- and supply-side activities on modern contraceptive use.

**Results:**

By endline, there was increased exposure to radio and television programming, religious leaders speaking favorably about contraception, and community-based initiatives. In the same period, modern contraceptive use increased from 16.9% to 22.1% with a slightly larger increase among the poor (16.6% to 24.1%). Multivariate analyses demonstrate that women exposed to community-based activities were more likely to use modern contraception by endline (marginal effect (ME): 5.12; 95% confidence interval (CI): 2.50–7.74) than those not exposed. Further, women living within 1 km of a facility with family planning guidelines were more likely to use (ME: 3.54; 95% CI: 1.88–5.20) than women without a nearby facility with guidelines. Among poor women, community-based activities, radio exposure (ME: 4.21; 95% CI: 0.49–7.93), and living close to program facilities (ME: 4.32; 95% CI: 0.04–8.59) impacted use.

**Conclusions:**

Community-based activities are important for reaching urban women, including poor women, to achieve increased contraceptive use. Radio programming is also an important tool for increasing demand, particularly among poor women. Impacts of other program activities on contraceptive use were modest.

**Implications:**

This study demonstrates that community-based activities led to increased modern contraceptive use among all women and poor women in urban Senegal. These findings can inform future programs in urban Senegal and elsewhere in francophone Africa.

## 1 Introduction

Prior research has demonstrated that both mass media programs and interpersonal communication activities generally lead to increases in knowledge, attitudes, discussion, and intentions to use family planning (FP), as well as actual use [1], [2], [3], [4], [5], [6]. Supply-side programs including quality of FP services and increasing access have also led to increases in knowledge, discussion, and in some cases, FP use [1], [7], [8], [9], [10]. Less is known about the impact of multi-component programs on FP outcomes [1]. Further, most previous evaluations focus on national-level programs, small-scale programs, or programs targeted to rural settings [1], [6]. Few evaluation studies have been undertaken in francophone Africa where recent analyses have demonstrated that there are gaps in uptake and use of FP [11]. This paper begins to fill these gaps by examining the impact of an integrated, multi-component FP program in urban settings in Senegal where at the time of program launch (2010), the modern contraceptive prevalence rate was only 20% based on Demographic and Health Survey data [12].

Using recently collected data from six cities of Senegal, this paper examines the impact on modern contraceptive use of the Senegal Urban Reproductive Health Initiative (called Initiative Sénégalaise de Santé Urbaine – ISSU) that included radio, television, community-based activities, religious-leader engagement, and improved quality of services. It considers impact among all women and poor women over a four-year follow-up period.

### 1.1 The Initiative Sénégalaise de Santé Urbaine

The ISSU project, led by IntraHealth International, was launched in 2010 with funding from the Bill & Melinda Gates Foundation (BMGF) with the goal of increasing use of modern FP in targeted urban sites. ISSU aimed to improve quality and integration of FP services, increase public-private partnerships and demand for FP methods, and advocate for increased funding for FP.

The ISSU project included demand generation activities as well as supply-side interventions. The first demand generation activity was working with Muslim religious leaders to become FP champions. The ISSU team trained Muslim religious leaders to promote FP messages in religious settings, in small group discussions, and to participate in debates about FP on the radio and television. Second, the ISSU program used radio, television, and the print media as part of their program activities. For example, ISSU included FP messages in various types of radio shows including health programs, religious programs, music programs, and through debates on the radio. They also undertook activities on specific, popular radio stations. Likewise, ISSU aired FP messages and advertisements on the television using specific stations that are popular in the study cities. There were also non-ISSU led national-level FP campaigns on the radio and television. The project undertook a small number of activities using print media including promoting FP through newspapers and magazines. ISSU also undertook community-based activities such as training community-health volunteers (called Badiénou Gokh) to share information about FP through one-on-one discussions with women and other members of the household or through small group discussions with multiple women in the home. Other community-level activities included: meetings where FP was raised and debated; conversations about FP that may have been led by a Muslim religious leader or another FP champion; educational discussions or awareness raising sessions (called “niche”) led by a community worker; and theater that raised issues related to FP.

The project also implemented supply-side activities in study cities. The first, and most extensive, of these activities was the Informed Push Model (IPM), a novel stocking and commodity tracking system that drew on private sector supply strategies to push commodities to facilities when they were at risk of depleting their stock rather than waiting for clinics/facilities to request stock when they were running low. ISSU implemented this innovation initially in two of the study sites (Pikine and Kaolack) and subsequently expanded nation-wide based on positive results in the initial sites. In addition, the ISSU program undertook activities to improve access to and quality of FP services, including: training providers; strengthening the referral system through training on the use of a systematic screening tool to identify client's FP needs; deploying midwives into facilities with gaps in services; and improving the private sector through pharmacy strengthening and support to Marie Stopes International in augmenting their FP services. The project was launched in 2010 in four initial urban sites: Dakar, Guédiawaye, Pikine, and Mbao with expansion in 2013 to two additional urban sites, Mbour and Kaolack.

At the same time that ISSU was funded, BMGF funded the Measurement, Learning & Evaluation (MLE) project that was tasked with designing and implementing a comprehensive impact evaluation of the ISSU project. The Carolina Population Center at the University of North Carolina at Chapel Hill led the MLE project.

## 2 Materials and methods

This study uses longitudinal data from women and facilities collected in 2011, prior to the start of ISSU program activities (baseline data), and 2015 (endline data) in all six cities. Longitudinal data for women allow us to use fixed effects methods that allow each woman to act as her own control [13]. At baseline, in each city, we collected data from a representative sample of women identified using a two-stage sampling design. In the first stage, we used the 2002 General Population and Housing Census's list of census districts (updated in 2009) as our primary sampling units (PSU) to select a random sample of PSU in each city. On average, census districts contain about 150–200 households. In total, we selected 268 PSU: 64 PSU from Dakar; 32 each from Guédiawaye, Pikine, and Mbao; and 54 each from Mbour and Kaolack. Prior to selection, we worked with municipal leaders to classify neighborhoods as poor and non-poor based on five characteristics: type of housing, residential security, neighborhood density, access to water, and access to flush toilets. Municipal leaders also gave an overall classification of the poverty level of the neighborhood. We weighted the five characteristics of neighborhoods (access to water had a weight of 2; toilets a weight of 1.5; and all others a weight of 1) and then summed. We considered those neighborhoods scoring in the lowest 40% as poor and all others as non-poor. Prior to selection, we stratified PSU by poor and non-poor and half of selected PSU were from the poor strata to increase inclusion of poor households and women. In the selected PSU, we listed/mapped all households and randomly selected 21 households for interview with equal probability [14].

In total, we selected 5628 households. In each selected household, all women of reproductive age (15–49) were eligible for interview. A trained female interviewer approached all eligible women and asked for their signed consent to participate. At baseline, we interviewed 9614 women across the six cities to provide a representative sample for each city at baseline. At endline, we tracked all baseline women who were usual residents (not visitors) of the household (*n*=9421) and, if found, asked them for consent to be re-interviewed.

The Institutional Review Board at the University of North Carolina at Chapel Hill and the Comité National d'Ethique in Senegal approved all study procedures.

Women interviewed at both baseline and endline are the analysis sample for examining the impact of the program on modern contraceptive use over the four-year follow-up period. We also undertake a sub-sample analysis of “poor” women, identified from the matched sample as those women who were in the two lowest wealth quintiles (poorest and poor women) at both baseline and endline.

At baseline and endline, we also collected data from health facilities in the study cities. We approached all public and private facilities offering reproductive health services (*n*=269) for interview. At each study facility, an interviewer administered a facility audit, provider interviews (up to four per facility), and exit interviews [15].

The key dependent variable for this analysis is use of modern contraception. At baseline and endline, trained interviewers asked women if they were using a contraceptive method to delay or avoid childbearing and if yes, interviewers asked what method the woman was using. Modern methods of contraception include male and female sterilization, daily pill, IUD, implants, injectables, male and female condoms, emergency contraception, Standard Days Method, and lactational amenorrhea. We coded women who reported traditional method use (e.g., rhythm method, withdrawal, or folkloric methods) as non-users. At each time point, we coded women who were abstinent or not sexually active as non-users.

This analysis examines the impact of exposure to various ISSU and non-ISSU demand generation activities and the ISSU and non-ISSU supply-side activities on the probability of modern method use. We classified the exposure variables as demand-side and supply-side. At endline, we included detailed questions on exposure to ISSU specific radio and television activities to assess the contribution of ISSU programming in target cities above and beyond the national-level radio and television programming (see Appendix Table A for details of questions used to measure exposure). We coded each of the exposure variables as dichotomous variables categorized as exposed versus unexposed. All exposure measures that were ISSU-specific and did not exist at baseline were coded zero (unexposed) at baseline.

We included four supply-side variables in this analysis. One of these is specific to the ISSU program: exposure to the ISSU Informed Push Model (IPM). To link the IPM variable to the women's data, we created a variable capturing whether the woman lived within 1 km of a facility with IPM at the time of the endline survey. This variable is coded zero at baseline since the IPM program activity did not exist at that time. We created three other supply variables based on this same 1 km buffer around where women live. These were: (a) woman lives within 1 km of a facility with any stock outs in the last 30 days; (b) woman lives within 1 km of a facility with a quality improvement committee; and c) woman lives within 1 km of a facility with observed FP guidelines.

We undertake descriptive analyses as well as multivariate analyses. We apply fixed effects regression to the pooled samples (baseline and endline) to reduce bias associated with self-selection, recall, and program targeting to underserved areas due to time invariant unobservables. Our estimation methods control for the possibility of endogenous attrition in the endline sample to the extent that attrition is due to unobserved fixed characteristics of the individuals. Fixed effect methods do not control for time varying unobservables. However, given the relatively close spacing of observations, time-varying unobservables are likely not a major source of residual bias in this setting. We estimated a classic fixed effects model whereby changes in modern contraceptive use depend on changes in time-varying individual characteristics and program exposure. All models control for marital status, age, education, city, religion, and wealth (contact corresponding author for full models). We perform two models, one including all women and the other for those in the two lowest wealth quintiles at baseline and endline. All analyses adjust for correlation at the community level using Huber-White type sandwich estimators for standard errors.

## 3 Results

The 9421 women surveyed at baseline were usual residents of the household and eligible for follow-up interview. Four-years later at endline, we found 6927 women, for an overall follow-up rate of 73.5% (see Table 1). Table 1 shows that women lost to follow-up were younger, more educated, in the poorest and richest wealth groups, not in union, and more likely to be from Dakar and Mbao; this can be seen in column 3 where there is a lower percentage interviewed at endline for each of these groups. The percentage followed-up was higher among modern method users (81%) compared to non-modern method users (72%); this relates to greater loss to follow-up among the younger women and women not in union who would be less likely to be using a method.

**Table 1:** Characteristics of women included in study samples for the Senegal impact evaluation at baseline (2011) and endline (2015), MLE

	Baseline†	Endline†	% Interviewed at endline by baseline characteristic
Characteristics	(*n*=9421)	(*n*=6927)	(*n*=6927); 73.5%
Age			
15–19	21.2%	3.9%	68.7%
20–24	21.5%	22.1%	69.2%
25–29	17.7%	21.2%	70.6%
30–34	13.6%	16.2%	77.4%
35–39	11.7%	13.5%	79.0%
40–44	8.6%	9.8%	82.9%
45+	5.7%	13.2%	83.3%
Education			
No education/Quranic	32.4%	31.3%	72.6%
Primary	33.4%	31.5%	75.6%
Secondary incomplete	20.0%	16.7%	76.3%
Secondary complete	8.3%	10.8%	69.3%
Higher	5.9%	9.7%	59.4%
Religion			
Christian	9.1%	9.4%	69.5%
Muslim	90.6%	90.4%	73.7%
None/other/missing	0.3%	0.2%	80.0%
Wealth quintile			
Poorest (1st quintile)	19.2%	21.7%	69.7%
Poor (2nd quintile)	19.5%	19.8%	75.0%
Middle (3rd quintile)	20.2%	19.2%	74.9%
Rich (4th quintile)	19.5%	20.3%	75.6%
Richest (5th quintile)	21.5%	19.0%	71.6%
Marital status			
Never married	40.8%	31.9%	68.3%
In union (at time of interview)	53.2%	60.4%	77.5%
Divorced, widowed, separated	6.0%	7.7%	69.8%
City			
Dakar	42.4%	41.4%	68.3%
Guédiawaye	10.8%	10.7%	73.3%
Pikine	11.3%	11.0%	75.4%
Mbao	21.7%	22.7%	67.6%
Mbour	6.3%	6.8%	79.9%
Kaolack	7.5%	7.6%	74.0%

† Each sample uses the weights from that sample and time period. Unweighted number of observations shown.

Table 2 presents women's use of contraception at baseline and endline among all women surveyed at both time periods and among those women who were in the two lowest wealth quintiles at both time periods (*n*=2038). Overall, modern method use increased from 17% at baseline to 22% at endline (p<.001) with a similar increase observed among poor women. There was also a transition in method mix between the two time periods. Implant use increased from baseline (9%) to endline (25%). This is paralleled by a decline between baseline and endline in short-acting methods like condoms (12% to 5%) and the pill (30% to 19%). Also shown in Table 2 are the transition patterns in women's use between baseline and endline. Overall, 70% of women remained non-users or traditional method users between baseline and endline. Another 8% transitioned from modern method use at baseline to non-use or traditional method use at endline. About 14% of women transitioned from non-users or traditional method users at baseline to modern method users at endline, and another 8% were modern method users at both time points.

**Table 2:** Women's contraceptive use at each time period, Senegal, MLE, 2011 and 2015

	All women		Poor women†	
	Baseline	Endline	Baseline	Endline
Contraceptive Use	(*n*=6927)	(*n*=6927)	(*n*=2038)	(*n*=2038)
Modern	16.9%	22.1%	16.6%	24.1%
Traditional	2.0%	2.3%	2.0%	2.6%
Non-user	81.1%	75.5%	81.4%	73.4%
Method mix (among method users)	(*n*=1269)	(*n*=1850)	(*n*=357)	(*n*=586)
Sterilization	2.2%	2.1%	1.3%	1.3%
Implants	8.6%	24.6%	13.6%	27.1%
IUD	5.8%	7.9%	3.5%	7.3%
Injectables	29.5%	31.2%	32.1%	33.1%
Pill	30.3%	19.2%	25.3%	15.7%
Male condom	11.6%	5.1%	12.7%	5.6%
Other modern methods†	1.4%	0.5%	0.9%	0.2%
Traditional method	10.7%	9.6%	10.7%	9.7%
Transitions in use between baseline and endline		(*n*=6927)		(*n*=2038)
Non-user or traditional user both times	na	70.1%	na	68.7%
Non-user or traditional user to modern user	na	14.1%	na	15.7%
Modern user to non-user or traditional	na	7.8%	na	7.3%
Modern user both times	na	8.1%	na	8.4%

Note: All percentages are weighted, sample sizes unweighted; sample includes women surveyed at both time periods. † Other modern methods include lactational amenorrhea, emergency contraception and female condom, spermicides, and Standard Days Method (endline only). † poor women include women in the two lowest wealth quintiles.

Table 3 presents the percentage of all women and poor women exposed to each of the program activities considered in this analysis. Between baseline and endline, the percentage of women exposed to FP in a newspaper or magazine, on the radio and television, and from a religious leader speaking favorably about FP increased. By endline 25.5% of all women and 29.5% of poor women reported exposure to ISSU led community-based activities such as community conversations, community meetings, and household visits by a community worker. Also presented in Table 3 is the percentage of women living within 1 km of a facility with stock outs, with a quality improvement committee, with FP guidelines, and with the ISSU supported IPM activity. Of note, more than 96% of women live within 1 km of an IPM/ISSU facility at endline and these facilities had reduced stock outs at endline as compared to baseline. Interestingly, there is a decline between baseline and endline for facilities having quality improvement committees and FP guidelines (i.e., 78% to 50% for all women for quality improvement). This may reflect changing facility environments where the guidelines or committees exist but the person interviewed at endline was not able to demonstrate their existence (i.e., show the document or a report from a committee meeting).

**Table 3:** Percentage of women exposed to ISSU and other program activities among all women and poor women, Senegal, MLE, 2011 and 2015

	All women		Poor women†	
	Baseline	Endline	Baseline	Endline
FP in Newspaper/magazine	4.4%	11.6%	2.8%	5.5%
Radio exposure to FP	19.8%	66.2%	19.7%	59.1%
Television exposure to FP	41.2%	92.5%	36.4%	85.7%
ISSU community religious talks about FP	0.0%	6.3%	0.0%	4.9%
Religious leader speaks favorably about FP	14.7%	57.5%	12.6%	54.6%
ISSU community activities	0.0%	25.5%	0.0%	29.5%
SMS messages about FP	0.9%	1.4%	0.4%	0.9%
FP on the Internet	0.0%	5.6%	0.0%	1.6%
Any stock outs in last 30 days for facilities w/in 1Km	68.3%	13.6%	72.1%	14.4%
Quality improvement committee at facility w/in 1Km	77.9%	49.8%	79.0%	54.0%
FP guidelines at facility w/in 1Km	72.2%	48.1%	71.5%	49.5%
ISSU/Informed Push Model (IPM) facility w/in 1Km	0.0%	96.1%	0.0%	97.4%

Note: All values weighted using the weights from the relevant time period. See Appendix A for description of variables used to create the exposure measures presented in this table.

† poor women include women in the two lowest wealth quintiles.

Table 4 presents the multivariate fixed effect impact results expressed as marginal effects in the pooled baseline and endline sample. Marginal effects (ME) indicate the average expected change in modern method use if each woman in the sample went from unexposed (to the particular program intervention) to exposed. The first model shows that the marginal effect (ME) of ISSU community-level activities is 5.12% (95% CI: 2.50–7.74; p<.001). Thus, if all women went from unexposed to exposed to community-level activities we would expect about a five percentage point increase in modern method use. In addition, women exposed to radio programs were also more likely to be using modern methods (ME: 2.17; 95% CI: −0.00 to 4.36) and women exposed to television programs were more likely to be using (ME: 1.69; 95% CI: −0.00 to 3.59), although these effects were only borderline significant (p=.052; p=.080, respectively). Further, if all women lived within 1 km of a facility with FP guidelines, we would expect a 3.54 percentage point increase in use (95% CI: 1.88–5.20; p<.001). Among poor women, we find that if all women went from unexposed to exposed to community-level activities we would expect an 8.47% increase in modern method use (95% CI: 3.98–12.97, p<.001). In addition, exposure to FP messages on the radio has a significant marginal effect at 4.21% (95% CI: 0.01–7.93; p=.027). Further, if all poor women went from not having an ISSU/IPM facility within 1 km to having ISSU/IPM services introduced to a facility within 1 km, there would be an increase of about 4.32% (95% CI: 0.00–8.59; p=.048) in modern method use. As for all women, if poor women went from having no facility within 1 km with FP guidelines to all women having a facility within 1 km with FP guidelines, we would expect modern contraception to increase by 2.96% (95% CI: −0.00 to 5.95; p=.053).

**Table 4:** Impact results, presented as marginal effects, of program activities on modern contraceptive use in Senegal, MLE, 2011 and 2015

	Marginal effects of 100% exposure	
	All women	Poor women†
	*n*=6927	*n*=2038
	% Change in CPR (SE %)	% Change in CPR (SE %)
Newspaper/magazine	−0.76 (1.86)	0.11 (5.02)
Radio exposure	2.17 (1.11)+	4.21 (1.89)*
Television exposure	1.69 (0.96)+	−1.23 (2.02)
ISSU community religious talks	1.55 (2.26)	−2.84 (4.17)
Religious leader speaks favorably about FP	0.72 (0.93)	1.56 (1.74)
ISSU community activities	5.12 (1.33)***	8.47 (2.28)***
SMS messages about FP	1.44 (4.15)	13.04 (9.98)
Internet	−2.35 (2.57)	−2.85 (8.69)
Any stock outs for facilities w/in 1Km	−1.71 (0.97)+	1.95 (1.83)
Quality improvement committee at facility w/in 1Km	0.05 (1.11)	−2.10 (2.00)
FP guidelines at facility w/in 1Km	3.54 (0.84)***	2.96 (1.52)+
ISSU/Informed Push Model (IPM) facility	−0.69 (1.16)	4.32 (2.17)*

+ p≤.1; * p≤.05; ** p≤.01; *** p≤.001. † poor women include women in the two lowest wealth quintiles.

Note: models were run as fixed effect linear regression using longitudinal baseline/endline data and control for age, marital status, religion, education, wealth group, and city of residence.

## 4 Discussion

This study employs longitudinal fixed effect regression methods to examine the impact of demand and supply-side activities on modern contraceptive use in six cities of Senegal. Our results demonstrate that community-based activities led to increases in contraceptive use observed by the follow-up period. The ISSU team implemented numerous types of community-level activities including training community-health volunteers (called Badiénou Gokh) to share information about FP through one-on-one discussions with women and other members of the household or through small group discussions with multiple women in their homes. Additional activities included community meetings where FP was raised and debated; community-based conversations about FP that may have been led by a Muslim religious leader or another FP champion; community-based educational discussions or awareness raising sessions (called “niche”) led by a community worker; and community theater that raised issues related to FP. The combination of these various community-level activities reached women in their homes or their communities. While only a quarter of women were exposed to community-based activities, the messages that were relayed were likely deeper than those exposed through the radio or television where the messages pass quickly and usually in a more generic format. That said, women exposed to the radio were also more likely to be using at endline, indicating the importance of this as a source of FP information as well. In particular, undertaking radio programming on stations that are commonly listened to and through debates and engagement of Muslim religious leaders on the radio may be important strategies implemented by ISSU to create demand for FP. Finally, supply-side activities including the existence of FP guidelines and for the poor, having an ISSU/IPM facility within 1 km, were related to increased modern method use. Lastly, we observe a shift among users towards more effective methods such as implants, IUD and injectables. This was at the expense of less effective, user-dependent methods such as the pill and condom.

Before concluding, a couple of limitations merit mention. First, Senegal has observed national level increases in modern contraceptive use over the follow-up period. The national-level urban contraceptive prevalence rate has gone from 20% in 2010–2011 [12] to 30% in 2015 [16]. This national-level increase demonstrates government commitment that is related to the rapid roll-out of the IPM to all public-sector facilities, reducing stock outs and increasing method choice for all women. Given that the public sector is the most common source of FP in Senegal, this increased availability likely had an impact on use that our models cannot account for. Second, we lost some women during the follow-up period. While our estimation methods correct for attrition related to unobserved time invariant characteristics of the women, some bias could be introduced due to unobserved time varying characteristics; with the data available, we cannot know how this potential bias affects the results.

To conclude, this paper demonstrates that large, multi-component urban targeted FP programming can lead to increases in modern method use in a context like Senegal that is part of francophone Africa and predominately Muslim. While initial FP use was low in this context and fertility desires were high, the multi-component program implemented by ISSU was able to reach women in their homes and increase modern contraceptive use and influence the method mix towards more effective methods. Program planners should consider these types of multi-component programs for other urban contexts of Senegal and elsewhere in francophone sub-Saharan Africa that have similar initial hurdles to FP use.

## Figures and Tables

**Table:** 

Category	FP Exposure Measure	Exposure Components
Demand	Newspaper or magazine	• Read about FP in a newspaper or magazine in the last 3 months
	Radio exposure	• Heard FP message on the radio in the last 3 months
		• Heard an FP spot/advertisement on the radio in the last year
		• Heard FP message on a health radio program in last year^1^^,⁎^
		• Heard FP message on a religious radio program in last year^2^^,⁎^
		• Heard FP message on a musical radio program in last year^3^^,⁎^
		• Heard FP message on an interactive radio program in last year^4^^,⁎^
		• Heard FP message on the radio program Xam sa yaram in last year^5^^,⁎^
	TV exposure	• Seen an FP message on the TV in the last 3 months
		• Seen an FP spot/advertisement on the TV in the last year
		• Heard FP message on Dine Ak Diamano on Walf TV in last year⁎
		• Heard FP message on Ndieguemar on Télévision Futurs Média (TFM) in last year⁎
		• Heard FP message on Li ci penc mi on TFM in last year⁎
		• Heard FP message on Thow li Thiow li on 2STV in last year⁎
		• Heard FP message on Sen DINE on la SEN TV in last year⁎
		• Heard FP message on Decriptage on la 2STV in last year⁎
		• Heard FP message on Xam sa yaram on Lamp FALL TV in last year⁎
	ISSU community religious talks	• Participated in the last year in a community-based religious talk
	Religious leader	• Have heard a religious leader speak favorably about FP in the last year (ISSU trained Muslim religious leaders as part of their program)
	ISSU community activities	• Heard FP message at a community meeting in the last year
		• Heard FP message at a community conversation in the last year
		• Heard FP message at a community based education session (niche anime) in the last year
		• Heard FP message at a home visit by outreach worker in the last year
	SMS messages about FP	• Received an FP message via SMS in the last 3 months
	Internet	• Saw an FP message on the internet in the last 3 months⁎
Supply	Stock-outs	• Woman lives within 1 km of a facility with any stock outs in the last 30 days
	Quality assurance committee	• Woman lives within 1 km of a facility with a quality assurance committee
	Family planning guidelines	• Woman lives within 1 km of a facility with observed FP guidelines
	ISSU and Informed Push Model (IPM) facility	• Woman lives within 1 km of an IPM/ISSU participating health facility

1 On the following radio stations: Rail Bi FM, Oxyjeunes, Afia FM, Mozdair, Jokko rufisque, dounya, Sud FM, Renndo, Lamp fall, Mbour FM or Alfayda.

2 On the following radio stations: Rail Bi FM, Oxyjeunes, Afia FM, Jokko rufisque, dounya, Sud FM KL, Renndo, Lamp fall, Mbour FM or Alfayda.

3 On the following radio stations: Rail Bi FM, Oxyjeunes, Afia FM, Jokko rufisque, dounya, Sud FM KL, Afia FM, Jokko rufisque, dounya, Sud FM KL.

4 On the following radio stations: Loci Xam sur Sud FM.

5 On the radio stations Lamp Fall FM.

⁎ Exposure was not measured at baseline for these items.
